# Bacteriophages and Bacterial Plant Diseases

**DOI:** 10.3389/fmicb.2017.00034

**Published:** 2017-01-20

**Authors:** Colin Buttimer, Olivia McAuliffe, R. P. Ross, Colin Hill, Jim O’Mahony, Aidan Coffey

**Affiliations:** ^1^Department of Biological Sciences, Cork Institute of TechnologyCork, Ireland; ^2^Moorepark Food Research Centre, TeagascFermoy, Ireland; ^3^Alimentary Pharmabiotic Centre, University CollegeCork, Ireland

**Keywords:** bacteriophages, plant diseases, biocontrol, biopesticides, phytopathogens

## Abstract

Losses in crop yields due to disease need to be reduced in order to meet increasing global food demands associated with growth in the human population. There is a well-recognized need to develop new environmentally friendly control strategies to combat bacterial crop disease. Current control measures involving the use of traditional chemicals or antibiotics are losing their efficacy due to the natural development of bacterial resistance to these agents. In addition, there is an increasing awareness that their use is environmentally unfriendly. Bacteriophages, the viruses of bacteria, have received increased research interest in recent years as a realistic environmentally friendly means of controlling bacterial diseases. Their use presents a viable control measure for a number of destructive bacterial crop diseases, with some phage-based products already becoming available on the market. Phage biocontrol possesses advantages over chemical controls in that tailor-made phage cocktails can be adapted to target specific disease-causing bacteria. Unlike chemical control measures, phage mixtures can be easily adapted for bacterial resistance which may develop over time. In this review, we will examine the progress and challenges for phage-based disease biocontrol in food crops.

## Importance of Crop Diseases

The human population is expected to reach 9.6 billion by 2050 and this will result in increased demands for food. It has been estimated that the global food supply may need to grow by as much as 70% in order to meet these demands (UN, 2013). For such growth, it has been predicted that crop supply may have to increase as much as 80–110% (Ray et al., 2013). To achieve these yields, the impact of crop disease has to be reduced. It has been estimated that at least 10% of global food production is lost to plant diseases (Strange and Scott, 2005). The major pathogens of plants are parasitic plants, oomycetes, nematodes, viruses, fungi and bacteria. Among the latter, there are over 200 plant pathogenic bacterial species (Considine and Considine, 1995). Those considered to be the most important belonging to the genera of *Pseudomonas, Ralstonia, Agrobacterium, Xanthomonas, Erwinia, Xylella, Pectobacterium*, and *Dickeya* (Mansfield et al., 2012).

## Bacteriophages, their Life Cycles and their Morphology

Bacteriophages (phages) are the most abundant biological entity in the biosphere with an estimated number of 10^31^, as total prokaryotic cell numbers are understood to be around 10^30^ in the biosphere and phage numbers are believed to be at least 10 times greater than this value (Whitman et al., 1998; Wommack and Colwell, 2000). Phages are specific viruses of bacteria that subvert the metabolism of their bacterial hosts in order to replicate. Of the phages that have been identified, the majority belong to the tailed phages; and these form the Taxonomic Order: *Caudovirales* (Ackermann, 2007). These phages possess icosahedral heads containing genomes comprised of double stranded DNA. The order *Caudovirales* is made up of three phage families; *Myoviridae* which have rigid contractile tails, *Podoviridae* with short, non-contractile tails and *Siphoviridae* with long flexible tails. Phages belonging to other families have highly variable morphologies with genomes of varying nucleic acid composition.

## History of Bacteriophages and their Use as Antibacterial Agents Toward Plant Diseases

The discovery of bacteriophages is credited to Frederick Twort (Twort, 1915) and Felix d’Herelle (d’Herelle, 1917). Similar findings of antibacterial agents that hinted on the existence of phage had been made prior to that of Twort and d’Herelle (Abedon et al., 2011). However, they were the first to suggest this phenomenon as being viral in origin. The potential of phages as antibacterial agents was quickly recognized, with d’Herelle in 1919 demonstrating the capability of his phage preparations to treat dysentery patients in the Hôpital des Enfants-Malades in Paris (Wilkinson, 2001). Following this work, many early studies and attempts were made to use phages to treat staphylococcal infections, cholera and bubonic plaque of humans (Sulakvelidze et al., 2001). This pre-antibiotic era approach became known as bacteriophage therapy. Studies were also initiated with the aim of using phages to control plant diseases. Mallmann and Hemstreet (1924) showed that the filtrate of decomposing cabbage could be used to inhibit the “cabbage-rot organism” *Xanthomonas campestris pv. campestris*. In 1925, Kotila and Coons demonstrated with bioassays that they could use phage to prevent soft rot by *Pectobacterium atrosepticum* and *Pectobacterium carotovorum* ssp *carotovorum* on slices of potato tuber and carrot, respectively (Coons and Kotila, 1925; Kotila and Coons, 1925). The first field trials were also done by Thomas (1935), who showed that he could reduce the incidence of Stewart’s wilt disease by treating seeds with phage against the phytopathogen *Pantoea stewartii* from 18% (untreated) to 1.5% (treated). However, this type of research became neglected as understanding of the nature of phage was poor at the time, and data on their efficacy was limited (Okabe and Goto, 1963).

## Bacteriophage Types Used for Therapy/Biocontrol

From a terminology perspective, the term bacteriophage therapy is usually reserved for human and animal applications. For plants the term bacteriophage biocontrol is more often used. In recent years, several studies have been published on phage biocontrol on a number of important bacterial plant pathogens, with many very promising results (see **Table 1**). The main deciding factor whether a phage is applicable for biocontrol (and also therapy in humans or animals) is whether a phage is exclusively lytic (virulent) or instead temperate in nature. Virulent phages are those which conduct infections that ultimately result in lysis of their host bacterium with the release of progeny phage particles. Temperate phages can follow the lytic route of infection but also follow the route of lysogeny, where the phage genome integrates into the bacterial chromosome or persists as a plasmid. In this form the phage is known as a prophage (Łobocka et al., 2004). With this strategy, the phage genome replicates as part of the bacterial genome of its host until a trigger switches it into the lytic cycle. These triggers can be chemical or physical (UV light or heat) in nature (Brunner and Pootjes, 1969; Müller et al., 2012). It is interesting to note that certain plant extracts can also trigger these events (Sato, 1983). Often, prophage DNA can increase the fitness of the bacterial host due to genes present on prophage genome. For example, in the case of plant pathogens, the *P. atrosepticum* prophages ECA41 and ECA29 both improve the motility of the bacterial host (Evans et al., 2010). Prophages may also harbor genes for toxins, e.g., shiga, cholera, and diphtheria toxins (Abedon and Lejeune, 2005). Another concern with these phages is the spread of virulence genes by transduction, where these phages can excise themselves from their host genomes incorporating host DNA into their own genomes facilitating horizontal transfer of genetic material among bacteria (Griffiths et al., 2000). Also, some lytic bacteriophages are capable of transduction, where they accidently pack bacterial DNA into their own capsid heads during the later stages of lytic cycle (Klumpp et al., 2008). There is also a third mechanism of phage-host interaction identified in filamentous phages (*Inovirus* family). Here, phages form a non-lethal chronic infection with continuous production of progeny phages. However, suitability of these phages for biocontrol is questionable as their infection can have varying effects on host virulence, as shown with phytopathogen *Ralstonia solanacearum* with its phage ϕRSS1 causing increased virulence (Yamada, 2013), although it has been shown possible to isolate virulent filamentous phage (Kuo et al., 1994). Another undesirable property in a phage intended for biocontrol is the ability to bring about superinfection exclusion to its host during infection. This prevents secondary infection of the host by another phage (Lu and Henning, 1994).

**Table 1:** Summary of bacteriophage biocontrol experiments which have been conducted since the year 2000 to the present.

Pathogen	Host	Disease	Information	Reference
*Pectobacterium carotovorum* ssp. carotovorum, *Pectobacterium wasabiae*, *Dickeya solani*	Potato	Soft rot	Bioassays with phage ΦPD10.3 and ΦPD23.1 could reduce severity of soft rot of tubers by 80% on potato slices and 95% with whole tubers from a mixed pathogen infection.	Czajkowski et al., 2015
*Dickeya solani*	Potato	Soft rot/Blackleg	Phage vB_DsoM_LIMEstone1 and vB_DsoM_LIMEstone2 reduced soft rot of inoculated tubers in bioassays and in field trials which produced a potato crop with higher yields.	Adriaenssens et al., 2012
*Dickeya solani*	Potato	Soft rot	Bioassays with phage ΦD1, ΦD2, ΦD3, ΦD4, ΦD5, ΦD7, ΦD9, ΦD10, ΦD11 could reduce incidence of soft rot by up to 30–70% on co-inoculated potato slices with pathogen and phage.	Czajkowski et al., 2014
*Streptomyces scabies*	Potato	Common scab	Seed tubers treated with phage Φ*A*S1 resulted in producing tuber progeny with reduced levels of surface lesion of scab (1.2%) compared with tubers harvested from non -treated seed tubers (23%).	McKenna et al., 2001
*Ralstonia solanacearum*	Tomato	Bacterial wilt	Tomato plants treated with phage ΦRSL1 showed no symptoms of bacterial wilt during the experimental period; whereas all untreated plants showed wilting 18 days post infection.	Fujiwara et al., 2011
*Ralstonia solanacearum*	Tomato	Bacteria wilt	Simultaneous treatment of phage PE204 with *R. solanacearum* of the rhizosphere of tomato completely inhibited bacterial wilt. However, pre-treatment with phage before the inoculation of pathogen was not effective with control of bacterial wilt, whereas post treatment of PE204 delayed disease development.	Bae et al., 2012
*Xanthomonas campestris pv. vesicatoria*	Tomato	Bacterial spot	Greenhouse experiments with formulated phage cocktails could reduce disease severity with formulated phage cocktails providing better protection in comparison to unformulated. A similar effect was found in three consecutive field trials.	Balogh et al., 2003
*Xanthomonas campestris pv. vesicatoria*	Tomato	Bacterial spot	In field experiments phage treatment was comparable to disease control with copper-mancozeb. Combination of phage and plant activator (ASM) resulted in enhanced control.	Obradovic et al., 2004
*Xylella fastidiosa*	Grapevines	Pierce’s Disease	*X. fastidiosa* levels in grapevines were significantly reduced on pre and post inoculation of a four phage (*Sano, Salvo, Prado and Paz)* cocktail. Pierce disease symptoms could be stopped using phage treatment post infection as well as applying phage prophylactically to grapevines.	Das et al., 2015
*Xanthomonas axonopodis* pv. *allii*	Onion	*Xanthomonas* leaf blight of onion	Field trial showed that weekly and biweekly applications of phage could reduce disease severity, a result which was comparable to treatments of weekly applications of copper-mancozeb.	Lang et al., 2007
*Pectobacterium carotovorum ssp. carotovorum*	Lettuce	Soft rot	Green house trials showed that phage PP1 could significantly reduce disease development on lettuce plants.	Lim et al., 2013
*Streptomyces scabies*	Radish	Common scab	Phages Stsc1 and Stsc3 could prevent disease development by treating radish seedlings. Non-treated radishes had 30% less weight than negative control, with phage treated radishes having masses similar to negative control.	Goyer, 2005
*Xanthomonas axonopodis* pv. *citri*	Grapefruit	Asiatic citrus canker	Five greenhouse experiments utilizing phage treatment could reduce disease severity by 59%. However, using a skim milk formulation of phage did not have increased disease control. Phage treatment was also capable of reducing disease occurrence in a citrus nursery. Control was less effective than copper-mancozeb. Combination did not give increased disease control.	Balogh et al., 2008
*Xanthomonas axonopodis pv. citrumelo*	Orange	Citrus bacterial spot	Phage treatments reduced citrus spot occurrence by 35 and 48% in two trials in commercial citrus nursery. Control was equal or less effective than copper-mancozeb. Combination did not give increased disease control	Balogh et al., 2008
*Pseudomonas syringae* pv. *porri*	Leek	Bacterial blight	Specific bio-assays demonstrated the *in planta* efficacy of phages vB_PsyM_KIL1, vB_PsyM_KIL2, vB_PsyM_KIL3, and vB_PsyM_KIL3b. However, phage cocktail of six phages (vB_PsyM_KIL1, vB_PsyM_KIL2, vB_PsyM_KIL3, vB_PsyM_KIL4, and vB_PsyM_KIL5 and vB_PsyM_KIL3b), were tested with two parallel field trial experiments in three locations which showed variable results. In one trial, symptom development was attenuated.	Rombouts et al., 2016
*Pseudomonas tolaasii*	Mushrooms	Brown blotch Disease	Surface of mushrooms were inoculated with pathogen. The formation of blotches was completely blocked by co-incubation of phages with pathogen.	Kim et al., 2011
*Erwinia amylovora*	Pear, apple trees	Fire blight	Phages ΦEa1337-26 and ΦEa 2345 reduced infection of detached pear tree blossoms by 84 and 96%, respectively, with *Pantoea agglomerans* as a carrier. Also, infection of potted apple tree blossoms could be reduced by 54% with phage ΦEa1337-26 and *P. agglomerans*. Control was comparable to streptomycin.	Boulé et al., 2011

Ideally a phage for biocontrol applications should be exclusively lytic and possess a host range which allows productive infection on all strains of the pathogen genus/species being targeted. Also, current opinion is that phages should be able to lyse the host quickly while producing high numbers of progeny phage and diffuse easily though the environment to which they are being applied. However, there was a report of a phage (ϕRSL1) of the phytopathogen *R. solanacearum* which was described as not highly lytic but still exhibited great biocontrol effect. The current standing theory of this phage’s disease prevention ability is that it is capable of co-existing without complete removal of its host from the soil surrounding crop roots, forming an equilibrium of infection that maintains the phage’s population but yet suppresses bacteria pathogenicity (Fujiwara et al., 2011).

While a given phage’s infection properties may appear to have great potential with *in vitro* studies, this does not necessarily translate into biocontrol potential in the field. Balogh (2006) showed in a study of three phages of *X. citri* pv *citri* exhibiting lytic activity in overlay plate assays that two of these phages were unable to lyse their host bacterium on grapefruit leafs, and indeed were later shown to be ineffective for the suppression of citrus canker in greenhouse trials. Attention should also be paid to the receptors that a given phage recognizes on a bacterial target. This can aid in the creation of phage mixtures with a reduced likelihood of host resistance (Frampton et al., 2014), and as such can lead to the development of phage combinations where individual members work in synergy to eliminate the target bacterium (Born et al., 2011).

## Advantages of Phage Biocontrol Over Other Strategies

Unlike chemical biocides, phages occur naturally in the environment and humans are thus exposed to them on a daily basis without any harm. After application, their numbers increase if their target bacterial host species are accessible to them. However, they tend to persist in high numbers in any environment only long as the host is present (Iriarte et al., 2012). Thus, phages are unlike copper-based pesticides which can potentially accumulate in the soil (Hirst et al., 1961; Pietrzak and McPhail, 2004). Phages generally have a narrow host range, typically being limited to stains within a particular species of bacteria. This can allow the creation of phage mixtures which can target bacterial species within a given genus of bacteria only. This could be a specific bacterial phytopathogen or it could be a particular bacterium in a microbial community whose suppression could help improve crop growth. Basit et al. (1992) for example, isolated phage which was unable to infect a desired strain of *Bradyrhizobium japonicum* which could aid soy bean crop growth due its nitrogen fixation properties, but could inhibit competing bacteria which did not possess this feature, thus allowing enhanced nitrogen fixation to occur.

Biofilm formation is an important factor in the virulence of phytopathogens such a *E. amylovora* (Koczan et al., 2011; Li and Wang, 2014). It is an attribute which has been shown to be involved in bacterial phytopathogen resistance to copper bactericides (Rodrigues et al., 2008). Phages have evolved to overcome this biofilm barrier through the use of depolymerase enzymes on their capsids but can also be released on host lysis, which allows them to degrade biofilm material, allowing the phage anti-receptor to gain access to the receptors on the surface of their host bacterium (Born et al., 2014). There is a growing demand by consumers for food produce that is free from chemicals biocides and preservatives. This has resulted in the restricted use of chemicals to produce “organic label” crops. The requirements of such food require the absence of chemical residues in crop production and processing (Lohr, 2001). Since phages are naturally occurring in the environment, they can be registered as biopesticides, making them suitable for more consumer-friendly organic farming (OmniLytics, 2006).

## Potential Issues Concerning the Use of Phage in Biocontrol

The main limitation for the application of phages in biocontrol in most settings is bacterial host-range. While this can be an advantage in certain circumstances, developing a phage-biocide that eliminates every member of a particular bacterial genus or species can be a challenge. Frequently the development of phage mixtures (cocktails) overcomes this disadvantage. Occasionally (but nevertheless, rarely) a phage is isolated which has an unexpectedly broad host-range. One example of this is a phage isolated from sewage and shown to target *Pectobacterium* and also enteric bacteria associated with humans (Pirhonen and Palva, 1988). Thus, careful attention should be given to ensure full understanding of likely host-range of a phage to avoid inefficacy or indeed to avoid the elimination of non-target potentially beneficial bacteria. In the latter context, instances of phage infecting beneficial bacteria resulting in reduced crop yield have been reported (Basit et al., 1992; Ahmad and Morgan, 1994).

It is believed that phages do not directly interact with plants. However, a number of phage-like genes have been identified in wheat, corn and *Arabidopsis* cress (Hedtke et al., 1997; Chang et al., 1999; Ikeda and Gray, 1999) which would suggest incorporation of phage DNA into the genomes of these crops and thus a possible a role in their evolution.

## Advantages of Phages in the Context of Host Resistance

Like antibiotics and copper sprays, for which resistance has been reported, there is also the possibility of bacteria becoming resistant to phage infection following constant exposure. However, unlike chemicals, phages are biological entities which can evolve and overcome these biological alterations in their hosts. There has always been a constant race between phage and bacteria in nature. This is indicated by the fact that 10–20% of bacterial populations in certain habitats are lysed daily because of phage infection (Suttle, 1994). In the context of phage resistance, Qiao et al. (2010) found that *Pseudomonas syringae* phage phi2954 was dependent on a host protein glutaredoxin 3 for successful infection. Mutant host strains without this protein were shown to be resistant to the phage. Nevertheless, these authors showed it was possible to isolate mutants of the phage that had become independent of this host protein for infection and this observation has been developed and employed in certain phages aimed at biocontrol. Flaherty et al. (2001) also showed that phages could evolve to overcome phage resistance in target bacteria and these were referred to as H-mutants. This allowed the development of phages with broader host ranges.

In addition to simple mutation-based phage resistance, bacterial phytopathogens can also possess other more complex resistance mechanisms such as the altruistic abortive infection (Abi) systems which give a bacterial host population immunity against a phage by causing phage-infected cells to commit suicide in order to prevent phage reproduction (Parma et al., 1992). While a number of these systems have been identified in *Lactococcus* starter culture strains found in dairy fermentations (Coffey and Ross, 2002; Chopin et al., 2005), recently such a system was identified in the phytopathogen *P. atrosepticum* and was termed ToxIN. This was characterized as a plasmid encoded Type III protein-RNA toxin-antitoxin system. The toxic protein ToxN is bound to RNA antitoxin ToxI in its inactive form. However, when phage infection occured, ToxI RNA antitoxin became unbound from ToxN causing death of the bacterial host cell (Fineran et al., 2009). Indeed, Blower et al. (2012) also showed using phage phiTE, that the phage was capable of creating mutants that could overcome this system by producing a pseudo ToxI RNA antitoxin preventing ToxN toxic activity.

Another mode of phage resistance is CRISPR/Cas systems, which are used by bacteria as well as archaea to form an immunity to protect from infection by foreign DNA such as phage. These systems are comprised of clustered regularly interspaced short palindromic repeat (CRISPR) arrays and CRISPR associated (Cas) proteins. In a recent study of 1,724 bacterial and archaeal genomes it was found that these systems were present in 10% of studied genomes. Previous studies had estimated CRISPR/Cas prevalence values of 40 and 80% of studied bacteria and archaeal genomes, respectively (Burstein et al., 2016). These have been detected in phytopathogens such as *P. atrosepticum* (Przybilski et al., 2011), *E. amylovora* (Rezzonico et al., 2011), and *Xantomonas oryzae* (Semenova et al., 2009). CRISPR arrays are comprised of short stretches of DNA (termed spacers), which are transcribed into short RNAs which interact with Cas proteins to detect and cut foreign DNA that match the sequence of the spacer (protospacer). Spacer sequences are acquired during exposure to foreign DNA in phage or plasmids, and thus they provide a genetic immunity from invasion by foreign DNA due to previous encounters (Marraffini and Sontheimer, 2008). However, it is also possible for phage to evolve to overcome these systems. Indeed, Semenova et al. (2009) detected a spacer in *X. oryzae* which matched a protospacer of phage Xop411. However, the phage was still able to infect this bacterium, due a mutation having occured in the protospacer sequence.

Bacteria developing resistance against phage infection is not necessarily a negative development in the context of phage biocontrol. Phage-resistance mutations in bacteria frequently are accompanied by a fitness cost, one example being a reduction in virulence, resulting in reduced disease severity. This results from the fact that molecules involved in phage attachment are frequently also involved in the virulence process. Examples include lipopolysaccharide (LPS) (Evans et al., 2010a), extracellular polysaccharide (EPS) (Ayers et al., 1979), flagella (Evans et al., 2010b; Addy et al., 2012) and pili (Ahern et al., 2014). Thus, mutations leading to resistance frequently compromise virulence. There, are however, a few examples where these mutations in bacteria surface structures did not lead to reduced virulence as seen with LPS production mutants of *Pectobacterium* and *Dickeya* (Schoonejans et al., 1987; Pirhonen et al., 1988).

## Bacteriophage and Chemicals

Phage have been shown to be stable in certain agrichemicals (Ravensdale et al., 2010). However, precautions need to be taken with some chemicals being combined with phage. Chemical biocides typically contain a range of phage inactivating substances such as surfactants and chelators (Yamamoto et al., 1968; Chattopadhyay et al., 2002). Also, copper-based bacteriocides have been shown to inactivate phage, but this inactivation can be avoided with the delayed application of phage (4–7 days) after initial application of copper-based bactericide (Iriarte et al., 2007).

## Complexity of Phage Interaction With Soil

The rhizosphere is the area of soil which is in close proximity to the roots of a plant. There are several factors which can affect phage activity in this environment such as pH, moisture levels, presence of organic matter and soil type. A number of these factors either individually or in combination can cause phage inactivation. Different soil types affect the survival of phage. For example, clay loam soils appear better at maintaining phage at low soil moisture levels and high soil temperatures than that of sandy loam soils (Straub et al., 1992) As well, low soil pH can also negativity affect phage survivability (Sykes et al., 1981).

Levels of adsorption of phage are affected differently in differing soil types, with levels of hindrance varying from one phage type to another (Goyal and Gerba, 1979). Phage can become bound to soil components such as clays (kaolinite and montmorillonite) as these minerals possess positively and negatively charged surfaces to which phage can adsorb (Schiffenbauer and Stotzky, 1982). Such adsorption can be influenced by pH (Goyal and Gerba, 1979; Loveland et al., 1996) as well as the presence of organic materials (Zhuang and Jin, 2003). Under favorable conditions, phages have been identified that persist at relatively stable concentrations for several weeks in soil (Fujiwara et al., 2011).

## Phage in the Phyllosphere

The phyllosphere is the portion of the plant which is above the ground and phages can readily be isolated from this location. How phages get there naturally has not been defined precisely, although it is possible that they originate in the soil from which the plant germinated - or alternatively get deposited by insect vectors. Indeed, phages for the phytopathogens *Pantoea stewartii* and *Erwinia herbicola* var. *herbicola* have been isolated from corn flea beetles (Woods et al., 1981). Another route is the translocation of phage from the roots to leaves of plants through the plant vascular system. And it has been shown that phages of *R. solanacearum, Xanthomonas perforans*, and *Xanthomonas euvesicatoria* can translocate though tomatoes plants, phage of *Xanthomanas oryzae* though the rice seedlings and phages of *E. amylovora* though apple seedlings and fire thorn (Rao and Srivastava, 1973; Iriarte et al., 2012; Kolozsváriné Nagy et al., 2015). However, this translocation may be influenced by the phage type, plant age, plant size, plant species, plant health and possibly soil type in which the plant is growing (Iriarte et al., 2012). It has also been reported that *E. amylovora* phages could pass from the leaves to the roots of apple seedlings (Kolozsváriné Nagy et al., 2015). The phyllosphere is nevertheless a harsh environment for phages to survive and it has been reported that their numbers can rapidly decline during daylight hours (Balogh et al., 2003; Iriarte et al., 2007). The destructive influence of UV light from the sun has been reported to be a limiting factor for the application of phages for successful biocontrol. The radiation causes the formation of lesions in DNA which can block DNA replication and transcription. In an vivo study with phage phiXV3-16, Iriarte et al. (2007) demonstrated a direct relationship between phage reduction on tomato leaves and increasing UVA+B dose. They also showed in an *in vitro* study that UV was capable of inactivating phage used against *Xanthomonas campestris pv. vesicatoria*, preventing it from exerting a biocontrol effect. Phage sensitivity against UV light has been shown to occur also with phage of *Dickya solani and E. amylovora* phages (Czajkowski et al., 2014; Born et al., 2015). However, there have been phages isolated against the phytopathogen *Pseudomonas syringae pv. actinidiae* which can tolerate extended UV-B doses (Yu et al., 2015). Other potential factors that could cause phage decline on the phyllosphere are desiccation, temperature, pH as well as certain chemicals produced by plants (Erskine, 1973; Delitheos et al., 1997; Iriarte et al., 2007).

## Phage Application Methods for Optimal Biocontrol Performance on Plants

One of the limitations to effective phage biocontrol on crops is the possibility of poor persistence on the phyllosphere due to the factors discussed in the previous section. However, several methods have been found to reduce this problem. Survival of phage can be improved in the phyllosphere and rhizosphere if they are accompanied by a viable host. This can be an avirulent strain of the pathogen being targeted or indeed another species of bacteria which occurs naturally in that environment (Svircev et al., 2006; Bae et al., 2012; Iriarte et al., 2012). It has also been found that avoiding daylight during application can improve phage-based biocontrol. Indeed, it has been demonstrated that applying phage to tomato leaves in the evening resulted in longer phage persistence in the phyllosphere, giving phage more time to infect and kill their bacterial targets (Balogh et al., 2003; Iriarte et al., 2007).

Born et al. (2015) conducted studies with number of substances to investigate if they gave phage protection against UV and reported that natural extracts from carrot, red pepper and beetroot all gave protection as did casein, soy peptone and also purfied aromatic amino acids, astaxathin and Tween 80. None of these subtances had a compromising effect on phage infection and stability (Born et al., 2015). Thus, it appears that a wide range of substances could enhance phage preformance in the phyllosphere with the main requirement being that they need to absorb UV thus limiting phage exposure. Biodegradable polymers have also been shown to give these protective effects (Khalil et al., 2016). In addition, Balogh et al. (2003) also showed an enhanced phage activity by combining the following preparations with phage, namely (i) 0.5% pregelatinized corn (PCF) and 0.5% sucrose, (ii) 0.5% Casecrete NH400, 0.5% sucrose and 0.25% PCF and (iii) 0.75% skim milk and 0.5% sucrose. These tests were performed in greenhouse trials and in field trials on tomato plants with phages against *Xanthomonas campestris* pv. *vesicatoria.* All formulations were used under a variety of different conditions, but generally demonstrated enhanced disease protection.

Soil based phage delivery is another approach that has been looked at to improve phage presistance in the phylosphere. Iriarte et al showed that a proprietary mixture of phage (OmniLytics Inc.) active against *X. perforans* strain 97-2 could translocate to the upper leaves of a tomato plant from its roots. They demonstrated that these phages which were applied to soil at levels of 10^8^PFU/mL could be detected at titres of 10^4^ PFU/g in leaf tissue for 7 days, whereas with a direct foliar application of the same phage mix, phage were undetectable 1 to 2 days after application (Iriarte et al., 2012). This work would suggest that the phage control of foliar plant diseases could be controlled by applying the phages to surrounding soil of a plant rather than by foliar spraying.

## Combination of Protective Methods Appear to be the Best Direction for Phage Biocontrol

There is evidence to support that combining phage with several methods used to control crop disease results in better control. The bacterium *Pantoea agglomerans* has been used as a biocontrol agent to suppress growth of the agent of fire blight *E. amylovora* and is being sold under the band name Bloomtime^®^ (Mikiciński et al., 2016). However, it has been reported that combining this bacterium with phage biocontrol can give enhanced protection that is comparable to that achieved with the antibiotic streptomycin (Svircev et al., 2006; Boulé et al., 2011). A similar observation of enhanced control was seen using a bacteriocin-producing strain of *R. solanacearum* with a phage to combat tobacco bacterial wilt (Tanaka et al., 1990). In another study, combining phage with Acibenzolar-S-methyl (ASM) was shown to have improved protection against bacterial spot of tomato in the field (Obradovic et al., 2004). However, combinations of phage with copper based pesticides do not appear to produce synergistic effects. Treatment with copper-mancozeb as seen with citrus canker and bacterial spot of citrus fruits did not produce synergy against *Xanthomanas axonopodis pv.citri* or *Xanthomanas axonopodis* pv. *citrumelo*, respectively (Balogh et al., 2008). As mentioned previously, this could be due to phage sensitivity to the components of these copper based sprays.

## Improved Understanding of Bacterial Host Diversity Should Aid Phage Biocontrol and Improve its Success in the Future

Recent years have seen recognition of the increasing diversity and complexity of bacterial phytopathogens mainly due to advances in molecular techniques (16S rRNA sequencing). For example, *X. campestris* pv. *vesicatoria*, which was previously a single species has since been divided into four (Jones et al., 2004). Another example is of the soft rot *Erwinia* group, which has undergone a significant taxonomic reshuﬄe with creation of novel species and genera (Hauben et al., 1998; Gardan, 2003; Samson et al., 2005). These developments are very important, as while the aﬄictions caused by these bacteria may appear identical on their respective crop targets, the phage sensitivities of the pathogens are likely to differ significantly, but nevertheless are likely to have some correlation with their taxonomic groupings. For example, the soft rot *Erwinia* group, which affects potato crops, has more recently been reclassified into two new bacterial genera (*Pectobacterium* and *Dickeya*), and these are relatively distinct from the point of view of phage susceptibilities (Czajkowski, 2016).

## Phytopathogens Targeted for Phage Biocontrol and how they are Currently Managed

There are a number of important bacterial plant pathogens that have received attention for phage biocontrol in recent years (**Table 1**) as existing approaches are having limited efficacy or their use is restricted in certain regions of the world. The following section discusses selected crop pathogens where phage biocontrol has been evaluated and is showing promise.

### *Dickeya* and *Pectobacterium*

Both *Dickeya* and *Pectobacterium* belong to the family of *Enterobacteriacea*, which collectively can be referred to as the Soft Rot *Enterobacteriacea* (SRE). Both genera characteristically produce several cell-wall-degrading enzymes that allow them to infiltrate and macerate the plant tissue on which they feed (Pérombelon, 2002). The plant host range of both bacterial genera is very broad: species belonging to *Dickeya* have been reported to infect 10 monocot and 11 dicot families, while those of *Pectobacterium* are reported to infect eleven monocot and sixteen dicot families (Ma et al., 2007).

*P. carotovorum* ssp *carotovorum* has a wide host range and global distribution, while *P. atrosepticum* is primarily found in temperate climates with a host range mainly limited to the potato (Pérombelon, 2002). *P. wasabie* and *P. carotovurum* ssp. *brasilensis* are also found to infect potato in several regions worldwide (Waleron et al., 2013; Lee et al., 2014). In Europe, *Dickeya dianthicola* is reported to be very important in potato disease, although more recently, a new *Dickeya* species called *D. solani* is being more frequently identified. Both also cause disease in other regions of the world (Toth et al., 2011). The economic impact of these potato infections can be severe. In the Netherlands, they cause annual losses in the seed potato sector of as much €30 million per year and in Israel, potato yield losses due to *Dickeya* have been as much as 20–25% (Prins and Breukers, 2008; Tsror (Lahkim) et al., 2008).

With regard to the potato, there are no effective bactericides to protect against SRE and the most effective approach has been through careful culturing practices, involving avoidance of contamination and the removal of diseased plants and/or diseased tissue. Certification systems are also employed. These involve the propagation of seed plants using healthy tissue culture plantlets followed by propagation in greenhouses, and then open field grow-out production. It is accompanied by careful monitoring and removal of diseased plants before release for general production. The generation number of these crops is also kept low to limit bacterial build up. However, the success of these certification schemes has been variable and heavily weather dependant (De Boer, 2004; Czajkowski et al., 2011).

### Erwinia amylovora

*Erwinia amylovora*, a member of the family of *Enterobacteriacea*, is the causative agent of fire blight which is a destructive disease that occurs to species of the plant family *Rosaceae.* The disease has been reported in 40 countries across North America, Europe, the Pacific Rim, and the Middle East (Bonn and van der Zwet, 2000). It heavily affects apple and pear production in several regions, with costs estimated as much as $100 million per year in the USA due to production losses and control measures (Norelli et al., 2003). It is considered to be a quarantine concern in countries belonging to plant protection agencies of APPPC (Asia and Pacific Plant Protection Commission), COSAVE (Comite Regional de Sanidad Vegetal para el Cono Sur), EPPO (Europe and Mediterranean Plant Protection Organisation) and IAPC (Inter-African Phytosanitary Council) (CABI, 2016).

Pathogenesis typically involves the bacterium entering a susceptible plant host though the nectarthodes of its flowers, but it may also enter the plant though other openings such as wounds (Bubán and Orosz-Kovács, 2003). Once in the plant, it is capable of moving though the intracellular space of parenchyma, where at the latter stages it may reach the xylem vessels. Under favorable conditions, disease can present itself as wilting, necrosis of tissue and dieback of the plant (Vanneste and Eden-Green, 2000). The bacterium does not produce cell-wall-degrading enzymes but the exopolysaccharide amylovoran, biofim formation capacity, motility, a type III secretion system, and quorum sensing are all understood to be features in its virulence (Piqué et al., 2015).

Traditionally, control of fire blight relies on cultural practices involving the removal of diseased tissue as well as preventative sprays containing copper or antibiotics (Norelli et al., 2003). However, issues with these chemical controls is copper tolerance of the pathogen and also the long term of use antibiotics (such as streptomycin) as a control strategy may be limited in the future, with growing concern of antibiotic resistance and the resulting restricted used of antibiotics for agriculture in certain regions of the world such as EU countries (Ordax et al., 2006; Russo et al., 2008; Mayerhofer et al., 2009; de León Door et al., 2013). As mentioned, biological controls using antagonistic bacteria have shown a capacity for controlling the disease (Mikiciński et al., 2016)

### Ralstonia solanacearum

*Ralstonia solanacearum* is a Gram negative soil-borne bacterium. It is considered to be one of most destructive phytopathogens with a host range of up to 200 plant species from over 50 families (Denny, 2007). The bacterium is highly heterogeneous, historically being divided into five races (based on plant host range) and five biovars (based on carbon utilization) (Denny, 2007). It causes diseases of economically important crops, such as bacterial wilt of tobacco, banana and tomato as well as brown rot of the potato (Sanchez Perez et al., 2008). The bacterium has global distribution (Sanchez Perez et al., 2008), and with regard to tomato and potato production, has quarantine status in the EU (Anonymous, 2000). The species has considerable economic impact: for example, brown rot of the potato has been estimated to exceed more than €950 million in losses per year worldwide (Scherf et al., 2010). Infection begins by the bacterium entering the host plant though its roots where it will then colonize the xylem. Infection typically leads to the development of yellowing of the plant, stunted growth, wilting and death, although the bacterium is also capable of asymptomatic infections (Sanchez Perez et al., 2008). Typical methods of control include the use of cultural practices such as selection of planting time, crop rotation, using clean seedlings and the use of resistant cultivars (Mariano et al., 1998). However, the use of such cultivars has shown a negative correlation between resistance and yields (Yuliar et al., 2015). Also, resistance possessed by these cultivars tends to be strain specific (Wang et al., 2000).

### Pseudomonas syringe

The bacterial phytopathogen *P. syringea* belongs to the class of *Gammaproteobacteria* (Hirano and Upper, 2000). The species is currently subdivided into more than 50 pathovars, with different pathovars representing different strains with differing plant host ranges (Hirano and Upper, 1990; Parkinson et al., 2011). Stains of most pathovars typically exhibit narrow host ranges, with pathovar P. *syringea* pv. *syringea* being an exception, having been reported to infect more than 80 plant species (Hirano and Upper, 2000).

*Pseudomonas syringea* pv. *tomato* causes necrotic lesions surrounded by a yellow chlorotic halos on tomato, a disease known as bacterial speck (Cruz et al., 2010). The pathovar can also infect members of genera of *Arabidopsis* and *Brassica* in laboratory setting (Elizabeth and Bender, 2007). The disease reduces yields while also affecting fruit quality (Fatmi, 2003). Pathogenesis by the bacterium involves the invasion of plant tissue from natural openings, such as stomata, where a type III secretion system plays a major role in its virulence with the release of effectors to overcome the plant immune system (Xin and He, 2013). It is spread by contaminated tomato seeds but can also survive as an epiphyte for extended periods on tomato plant surfaces and is dispersed in windblown rain (Smitley and McCarter, 1982; McCarter, 1983; Preston, 2000). Control of the organism typically involves the use of uncontaminated seeds and the used of bactericides (copper and streptomycin) to limit its spread (Preston, 2000; Fatmi, 2003). However, copper tolerant strains of the bacterium have been reported (Alexander et al., 1999).

### *Xanthomonas* species

*Xathomonas* is a large genus, which belongs to the class of *Gammaproteobacteria*, containing at least 27 official species, many of which also possess several pathovars. Collectively, the genus host range is broad: infecting around 400 plant hosts, a number of which are important crops such rice, banana, tomato, and citrus fruits. Species and pathovars of this genus typically exhibit a high degree of host- as well as tissue-specificity, invading either the xylem or intercellular spaces of the mesophyll parenchyma tissue (Ryan et al., 2011).

*Xathomonas campestris* pv. *vesicatoria* is the causitive agent of bacterial spot disease of tomato and pepper, with the disease having been identified in many countries worldwide (Jones et al., 2005). This tomato disease can be very severe with yield losses of up to 50% reported for tomatoes grown both in greenhouses and fields in the USA and Caribbean (Camesano, 2015). Disease is caused by the bacterium entering the plant though stomata or wounds. The bacteria then colonize the intercellular space of the plant, inducing water-soaked lesions that later become necrotic, which can result in defoliation and severely spotted fruit (Thieme et al., 2005). Control of the disease has involved preventative cultural practices such as avoiding unnecessary crop damage and using uncontaminated seed, but also includes use of resistant cultivars as well as chemical controls with copper or streptomycin (Goode and Sasser, 1980). However, the use of resistant cultivars has not always been successful and there have been reports of bacteria developing resistance to the above two agents (Goode and Sasser, 1980; Ritchie and Dittapongpitch, 1991; McDonald and Linde, 2002).

### Xylella fastidiosa

*Xylella fastidiosa* belongs to the of class of *Gammaproteobacteria.* It is a xylem-limited phytopathogen that requires insect vectors (such as sharpshooters) for its distribution and infection of its host plants (Chatterjee et al., 2008). It causes disease on a number of crops such as the grape, citrus, almond, peach and coffee (Hopkins and Purcell, 2002). While it has primarily been contained in the Americas, it has been indentified in Europe in recent years causing disease on olive trees (Hopkins and Purcell, 2002; Loconsole et al., 2014). Disease caused by the bacterium is believed to be induced by the formation of biofilm aggregates in the vascular system, which restricts the movement of nutrients and water throughout the plant (Chatterjee et al., 2008). It causes Pierce disease of the grapevine, a highly destructive infection, which heavily affects grape production in the USA, and has been estimated to cost as much as $104.4 million annually to the state of California (Tumber et al., 2014). Existing control methods have been limited in their management of the disease and include removal of infected plants and control of the infected insect vector populations with neonicotinoid-based insecticides (Janse and Obradovic, 2010). However, the use of these insecticides has seen restrictions in recent years due to their possible effects on honey bee populations (Anonymous, 2013; Lu et al., 2014).

## Critical Summary of Recent Phage Biocontrol Studies on Crops

There is growing evidence showing that phage have promising biocontrol applications for number of plant diseases in different crops. The following section describes recent studies that have been conducted since the year 2000 and the findings from these is summarized in **Table 1**.

The most common crops that appear to benefit from the application of phages for biocontrol in recent scientific literature are the potato and the tomato, as both have been the focus of numerous recent studies. The bacterial pathogens in the case of the potato are predominantly the SRE. As mentioned above, one of the most important SREs in Europe is *D. solani;* and the potential of phage to control this phytopathogen have been assessed indicating strong potential for disease control. For example, Adriaenssens et al. (2012) conducted a bioassay and a field trial using phage (LIMEstone1). The bioassay involved the incubation of seed tubers (cultivar Bintje), which had either been inoculated with the bacteria or co-inoculated with the bacteria and the phage (MOI of 100). They showed that tubers inoculated with the bacteria alone would experience to 40% maceration of tuber tissue, while those co-inoculated with the phage and bacteria exhibited no more than 10% maceration of tuber tissue. Similar results were observed with the seed tuber cultivar Kondor. The field trial using the same phage against the same pathogen also suggested it was capable of exerting this biocontrol effect *in-planta*, as phage treated infected seed potatoes resulted in higher crop yields than those without phage treatment. Similar findings were reported by Czajkowski et al. (2014) who also isolated phages specific for *D. solani*. These workers conducted bioassays with tuber slices incubated with the bacterial pathogen with or without phages (MOI of 0.01) and showed that the application of phages could prevent potato tuber tissue maceration by up to 70%. SREs other than *D. solani* were also studied for their susceptibility to phages by the same group. They found that the application of phages (MOI of 0.01) to control *P. carotovorum ssp carotovorum* and *P. wasabie* destruction could prevent damage of up to 80% on tuber slices and up to 95% on whole tubers against tissue maceration from a mixed bacterial infection (Czajkowski et al., 2015). Such data is highly encouraging as many SRE infections tend to result for a mixture of genera/species. Aside from potato, SRE infections have also been controlled by phage in lettuce, with high levels of disease prevention being reported (Lim et al., 2013). Aside from the SRE problem, potato infections from the Gram-positive bacterium *Streptomyces scabies* results in the formation of a corky lesion (known as common scab) on the tuber and indeed other root vegetables also, as well as causing the reduced growth of seedlings (Lerat et al., 2009). This pathogen has also been successfully treated in potato by phage biocontrol and thus has implications for other crops also as demonstrated by Goyer (2005). In conclusion, the above studies indicate strong potential for phage based control of these diseases.

Another crop which has been the focus of several studies in the context of phage therapy is the tomato, which is commonly infected by *R. solanacearum* (also causes brown rot in the potato) and *X. campestris* pathovars. Again, phage biocontrol approaches have been demonstrated to give a significant reduction in bacterial wilt (*Ralstonia*) and leaf spot caused by *Xanthomonas*. Indeed, the successful trials against *R. solanacearum* reported by Mansfield et al. (2012) are significant considering the wide host range of the bacterium. Similarly, in the case of *Xanthomonas*, the observed beneficial effect of the application of phages can also be extrapolated to other plants affected by pathogens belonging to the same genus. Indeed, studies on elimination of *Xanthomonas* using phage have been conducted with successful outcomes on both grapefruit and orange (Balogh et al., 2008) as well as onion (Lang et al., 2007). A variety of other crop infections have also been reduced in severity in other phage biocontrol studies. These include *Pseudomonas* infections of mushrooms (brown blotch) and leeks (bacterial blight) and infection of the grapevine by *Xylella* (Das et al., 2015).

## Commercialization of Phage for Biocontrol in Crop Disease

In recent years, several phage biocontrol products have reached the market. A USA based company Omnilytics was the first company to receive registration (from the US Environmental protection agency) for their phage based biopesticide product Agriphage. The product is designed for the control of bacterial spot or speck of tomatoes and peppers (specific for *X. campestris* pv. *vesicatoria*, or *P. syringae* pv. *tomato*). This product has also received an OMRI listing making it suitable for use by commercial organic growers (OmniLytics, 2006). A Hungarian company Enviroinvest was the second company to receive registration for their biopesticide named Erwiphage for the control of fire blight of apple trees (specific for *Erwinia amylovora*) (Enviroinvest, n.d.). There is also a Scottish company, APS biocontrol, which has developed a bacteriophage-based wash solution (Biolyse) for potatoes tubers, which is to be used for prevention of soft rot disease (specific against soft rot *Enterobacteriacea*) during storage (APS Biocontrol Ltd, n.d.). Interestingly this product has been reported to be used by the Tesco supermarket chain (Branston, 2012).

However, in some regions of the world there are delays that have to be overcome with regard to legislation allowing phage biocontrol approaches for the of control of bacterial plant diseases. A problem with phage-mediated biocontrol is that phage mixtures/cocktails need to be updated constantly in order to lyse as many newly emerging strains of the target bacterium as possible. This approach is used by Omnilytics (OmniLytics, 2004). This allows a phage cocktail to be adapted to the relevant disease-causing bacterial strains in a given situation, also facilitating counteraction of any phage resistance development during the phage application. However, EU regulations (1107/2009 EC) require that any change to one of the components of a phage cocktail would require reregistering which requires time and expense, making the US approach currently unfeasible in the EU (Doffkay et al., 2015). Legislation governing phage biocontrol may need to become more malleable in the EU for the best application and performance of phage products as biopesticides.

## Other Phage Applications of the Past and Possible Future with Regards to Phytopathogens

Phage typing schemes have been employed for several phytopathogens for epidemiology studies (Toth et al., 1999; Ahmad et al., 2014). These systems allow the identification of a particular strain of a species based on their susceptibility to series of phage. The downfall of this method, however, is that it depends on the isolation of pure cultures for identification as well as the maintenance of stocks of typing phage as well as host strains for which to propagate them. Nowadays, studies of phytopathogens has moved away from phage typing due to its tendency to generate false positives and false negatives results as well as its low resolution and the development of new and improved molecular techniques (Czajkowski, 2016)

Several phage-based detection systems have been developed for human and animal pathogens (van der Merwe et al., 2014). Recently however, work has been published on the promising application of these methods for the detection of plant pathogens. Such a detection system has been developed for *R. solanacearum*, which is based on detection of the bacterium by phage propagation followed with quantitative PCR (qPCR). Samples that contain the bacterium will cause added phage titres to increase, these titre increases can then be detected using qPCR. This method was found to be faster than conventional methods with greater sensitivity allowing detection of 10^2^ CFU/g of soil, 10^3^ CFU/ml from drainage water from potted plants and 10^2^ CFU/g in 0.1 g of leaf tissue. The method also does not require the destruction of a plant for the detection of bacterium unlike those currently used to detect *R. solanacearum* (Kutin et al., 2009). It is possible to engineer phage of phytobacteria into reporter systems that can emit a detectable bioluminescent signal during infection. A “*luxAB-*tagged” reporter phage was developed for *Pseudomonas cannabina pv. alisalensis* (agent of bacterial blight of crucifers) which was shown capable of detecting the bacteria within minutes. This phage was also capable of emitting a detectable signal during infection of both cultures and diseased plant samples (Schofield et al., 2013). Both the mentioned systems have advantages over other molecular detection methods, in that phage propagation requires active metabolism, conveniently limiting it to viable bacterial cells.

## Conclusion

Effective control of plant disease typically calls for a disease management strategy that involves several integrated approaches. Currently, the use of phage biocontrol is an emerging, but as yet uncommon practice. However, phages do possess several properties which can add to the arsenal of controls for crop diseases. They are natural, making them suitable for organic farming. They can be used to create phage cocktails with tailored host ranges. Also, phages naturally have the potential to evolve to adapt to overcome phage-resistance or overcome new strains of bacteria. They can be combined with other chemical or biocontrol agents. A possible limitation to their use is their sensitivity to UV light and to certain soil conditions. However, approaches have been found to overcome some of these limitations with the use of UV protectant formulas and timing of the application of phage to crops to avoid interaction with chemical pesticides and exposure to UV light. In addition to biocontrol applications, there is also good potential for phage-based diagnostics for plant pathogenic bacteria with a high sensitivity aimed specifically at viable bacteria.

Many pesticide companies are moving away from investment in chemical pesticides and increasingly directing their attention to biopesticides. The pesticide market is worth $56 billion with the biopesticides forming only $2–3 billion of this. However, growth of the biopesticide sector is expected to outpace chemical pesticides in the future (Marrone, 2014). This change is believed to be due to an increasing customer demand for chemical residue free foods and increasing legalization on the use of synthetic pesticides in certain regions in the word. In addition, many biopesticide products are potentially cheaper to develop and quicker to bring to the market (Marrone, 2014). With this economic environment, one can expect to see increased activity in the development of phage biocontrol as a viable approach for crop disease control in the future.

## Author Contributions

CB wrote this article. OM, RR, CH, JO, and AC critiqued and provided direction towards the article. AC is financing the publication.

## Conflict of Interest Statement

The authors declare that the research was conducted in the absence of any commercial or financial relationships that could be construed as a potential conflict of interest.
